# Sex Differences in All-Cause and Cardiovascular Mortality During Long-Term Follow-Up in Patients with HFrEF

**DOI:** 10.3390/jcm15218431

**Published:** 2026-10-29

**Authors:** Andrea Lopez-Lopez, Estefanía Rivas Vázquez, Carmen Cristina Álvarez-Suárez, Margarita Regueiro-Abel, Charigan Abou Johk-Casas, Juliana Elices-Teja, Ramón Ríos Vázquez, Eva María Pereira López, Germán Santamarina-Pernas, Carlos González-Juanatey

**Affiliations:** 1 Department of Cardiology, Hospital Universitario Lucus Augusti, 27002 Lugo, Spaincarlos.gonzalez.juanatey@sergas.es (C.G.-J.); 2 CardioHULA Research Group, Instituto de Investigacion Sanitaria de Santiago de Compostela IDIS, 27002 Lugo, Spain; estefania.rivas.vazquez@sergas.es; 3 Department of Anatomy, Animal Production and Veterinary Clinical Sciences, Santiago de Compostela University, 27001 Lugo, Spain; 4 Institute for Research in Global Health and Sustainable Development (iTERRA), Campus Terra, Universidad de Santiago de Compostela, 27002 Lugo, Spain; 5 Nursing School, Santiago de Compostela University, Campus Lugo, 27001 Lugo, Spain

**Keywords:** heart failure, sex characteristics, mortality, competing risks, prognosis, survival analysis

## Abstract

**Background/Objectives**: Sex-related differences in prognosis among patients with heart failure with reduced ejection fraction (HFrEF) have been described, but their association with long-term all-cause and cardiovascular mortality remains incompletely characterised. This study investigated these associations in a real-world HFrEF cohort, accounting for non-cardiovascular death as a competing event. **Methods**: We conducted a single-centre, retrospective, longitudinal observational study of 1510 consecutive patients with HFrEF followed between 2011 and 2023; 1158 (76.7%) were men and 352 (23.3%) were women. All-cause mortality was assessed using Kaplan–Meier methods and Cox regression. Multivariable models used a complete-case approach. Cardiovascular mortality was analysed using cause-specific Cox regression and the Fine–Gray model, with non-cardiovascular death and death of unknown cause treated as competing events. Interaction terms between sex and year of inclusion were used to assess potential changes in the association across the study period. **Results**: No statistically significant between-sex difference in cumulative all-cause mortality was detected at one year (3.7% in men vs. 3.8% in women; *p* = 0.95). Exploratory pointwise comparisons showed higher cumulative mortality in men at five years (24.8% vs. 16.0%; *p* = 0.002) and ten years (49.3% vs. 39.8%; *p* = 0.046). After multivariable adjustment, male sex was associated with higher all-cause mortality (HR, 1.95; 95% CI, 1.47–2.59; *p* < 0.001). Male sex was also associated with cardiovascular mortality in the cause-specific analysis (HR, 2.46; 95% CI, 1.54–3.92; *p* < 0.001) and after accounting for non-cardiovascular death as a competing event (subdistribution HR, 2.12; 95% CI, 1.32–3.39; *p* = 0.002). No significant interaction was observed between sex and year of inclusion. **Conclusions**: In this real-world HFrEF cohort, male sex remained associated with higher all-cause and cardiovascular mortality after adjustment for measured baseline covariates. The association with cardiovascular mortality persisted in competing-risk analysis. These observational findings do not establish a causal or time-varying effect of sex.

## 1. Introduction

Despite substantial advances in the treatment of heart failure with reduced ejection fraction (HFrEF), mortality remains high [1]. Prognosis in HFrEF is heterogeneous and is determined by a complex interplay of clinical characteristics, comorbidities, disease aetiology, and changes in cardiac function over time [2]. Identifying factors associated with mortality therefore remains essential for improving risk stratification and understanding the clinical course of patients with HFrEF.

Importantly, the prognostic profile of HFrEF is not static. The relative contribution of individual risk factors may change throughout the course of the disease, with different determinants becoming relevant during early and late follow-up and according to the underlying cause of death. Our group previously demonstrated that predictors of mortality displayed a time-dependent pattern in a cohort of HFrEF patients: renal function and ischaemic aetiology were particularly relevant to early mortality, whereas age, hypertension and, notably, changes in ventricular function became increasingly important during long-term follow-up [2]. These findings suggest that the prognostic significance of clinical characteristics may evolve over time and raise the possibility that sex may also be associated with mortality differently according to the stage of follow-up and the cause of death.

Sex is a potentially important, yet incompletely characterised, determinant of prognosis in HFrEF. Evidence informing the treatment of HFrEF has historically been derived predominantly from male populations. Women have consistently represented approximately 25% of participants in HFrEF clinical trials, with little improvement in their representation over the past two decades [3]. Women also remain under-represented in trials supporting contemporary clinical guidelines [4]. Beyond this disparity in clinical research, men and women with HFrEF differ in disease characteristics, comorbidity profiles and potential underlying mechanisms [5,6]. Men more frequently develop HFrEF of ischaemic origin, whereas women tend to be older and more frequently have non-ischaemic aetiology, poorer renal function and a greater symptom burden [6,7]. Biological differences, including sex-related patterns of ventricular remodelling and the effects of sex hormones, may further contribute to differences in disease progression and outcomes [8]. In addition, women may be less likely to receive optimal guideline-directed medical therapy and appropriate treatment-dose escalation [9]. Together, these phenotypic, biological and treatment-related differences provide a rationale for investigating whether sex is associated with distinct long-term outcomes in HFrEF.

Several studies have reported lower mortality among women with HFrEF [10,11]. In a pooled analysis of the PARADIGM-HF and ATMOSPHERE trials, Dewan et al. reported lower overall and cardiovascular mortality in women after multivariable adjustment [10]. Similarly, data from the KorAHF registry showed that male sex was independently associated with higher overall and cardiovascular mortality among patients with HFrEF [11]. These findings support an association between sex and prognosis in HFrEF; however, whether this association remains constant throughout follow-up is less well established.

Accumulating evidence suggests that the prognostic association of sex may be time-dependent. In a meta-analysis including more than 700,000 patients, Qiu et al. found that the lower mortality observed in women was more pronounced among patients with chronic heart failure and in studies with follow-up periods longer than 2 years [12]. Similarly, Klempfner et al. reported no significant difference in mortality between men and women during the first 6 months after hospitalization for heart failure, whereas male sex was associated with higher mortality during subsequent follow-up [13]. Other studies have also explored sex-related differences according to follow-up duration, left ventricular ejection fraction, or cause of death, although findings have not been fully consistent [14,15]. Taken together, these observations suggest that the association between sex and mortality may depend not only on patient characteristics but also on the temporal horizon and the specific mode of death considered.

The distinction between cardiovascular and non-cardiovascular mortality is particularly relevant in this context. Non-cardiovascular death represents a clinically important competing event in patients with HFrEF because its occurrence precludes subsequent cardiovascular death [1,16]. Accordingly, conventional time-to-event approaches that treat competing events as non-informative censoring may overestimate the cumulative incidence of the event of interest. Competing-risk methods, including cumulative incidence functions, allow the probability of a specific cause of death to be estimated while accounting for the occurrence of alternative causes of death [17]. Incorporating competing risks is therefore important when assessing whether the association between sex and mortality differs according to the cause of death.

Although predictors of early and late mortality have previously been investigated in this cohort [2], the present analysis focuses on sex-related differences in long-term all-cause and cardiovascular mortality. Its additional contribution is the assessment of cardiovascular mortality using complementary cause-specific and competing-risk models. Accordingly, we investigated the association between sex and these outcomes and assessed whether it differed according to calendar year of inclusion or ischaemic aetiology.

## 2. Methods

### 2.1. Study Design and Patients

This was a single-centre, retrospective, longitudinal observational study of patients with heart failure with reduced ejection fraction (HFrEF) followed at the Heart Failure Unit of the Cardiology Department of University Hospital Lucus Augusti, Lugo, Spain. The study included the same cohort of 1510 consecutive patients previously described [2], whose follow-up at the unit began between January 2011 and December 2023.

The index date was defined as the date of the first consultation at the specialist Heart Failure Unit. Patients were referred through three main pathways: following hospital discharge, from other outpatient clinics after an initial diagnosis of HFrEF, or after an emergency department visit for acute heart failure decompensation. Baseline clinical, laboratory, and echocardiographic variables were recorded at this initial specialist assessment.

Eligible patients were aged 18 years or older and had a documented diagnosis of HFrEF, defined as a left ventricular ejection fraction (LVEF) ≤40% at the index visit. Patients with an LVEF >40% at the time of entry were excluded. Both patients with stable chronic heart failure and those referred after stabilization from an acute decompensation were included, irrespective of heart failure etiology. Accordingly, patients with ischemic cardiomyopathy, severe valvular disease, and relevant comorbidities, including active cancer, advanced chronic kidney disease, or maintenance dialysis, were eligible.

Patients without available survival information were excluded from survival analyses. For multivariable analyses, a complete-case approach was applied, excluding patients with missing data for any covariate included in the corresponding model.

For this study, the cohort was analysed by sex, comprising 1158 men and 352 women.

### 2.2. Data Collection and Definitions

Clinical, demographic, laboratory, echocardiographic, therapeutic and mortality data were obtained by reviewing the electronic health records. The variables collected included age, sex, cardiovascular risk factors, the aetiology of heart failure, comorbidities, the New York Heart Association (NYHA) functional class and renal function, assessed using estimated glomerular filtration rate.

Sex was recorded in the clinical database as a binary variable (male or female), reflecting routinely documented biological sex. Information on gender identity and related sociocultural characteristics was not collected. To characterise any potential baseline differences between the two groups, the following variables, amongst others, were analysed: age, body mass index, renal function, NYHA functional class, aetiology of heart failure, cardiovascular risk factors and major comorbidities. Clinically relevant variables were subsequently considered as potential confounding factors in the multivariable models.

Left ventricular ejection fraction (LVEF) was assessed by echocardiography at the start of follow-up. In the previous study of this cohort, echocardiographic assessment was performed using IE-33 and EPIQ-5 systems (Philips, Bothell, WA, USA).

Baseline pharmacological treatment was also recorded. For the present analysis, specific consideration was given to the main therapeutic classes used in HFrEF: beta-blockers, angiotensin-converting enzyme inhibitors/angiotensin receptor blockers/angiotensin receptor-neprilysin inhibitors (ACEi/ARB/ARNI), mineralocorticoid receptor antagonists (MRA) and sodium-glucose cotransporter 2 inhibitors (SGLT2i). Treatment with loop diuretics, statins and anticoagulants was also recorded.

As the inclusion period spanned from 2011 to 2023 and coincided with significant changes in the treatment of HFrEF, the year of inclusion for each patient was also recorded.

### 2.3. Follow-Up and Outcome Variables

Follow-up began on the date of the first consultation at the Heart Failure Unit. Vital status was ascertained individually for each patient using the most recent available follow-up information. Follow-up began at the first consultation at the Heart Failure Unit and continued until death, 10 years of follow-up, or the last available assessment of vital status, whichever occurred first.

The outcome variables analyzed were all-cause mortality and cardiovascular mortality. Cumulative all-cause mortality was assessed over a follow-up period of up to 10 years, with specific estimates at 1, 5 and 10 years, to describe between-sex differences in cumulative mortality at selected follow-up time points.

Mortality events and their causes were reviewed and validated by the researchers through the assessment of electronic medical records, hospital discharge reports, A&E records and primary care documentation, following the same criteria used in the previous study of this cohort [2]. Cardiovascular mortality was defined as death due to progression of heart failure, sudden cardiac death, acute coronary syndrome or stroke. Deaths from malignant neoplasms, infection or sepsis, severe functional deterioration or other non-cardiac causes were classified as non-cardiovascular mortality. Where the exact cause could not be determined from the available documentation, it was classified as unknown. For analytical purposes, non-cardiovascular deaths and deaths of unknown cause were combined into a single competing-event category. Accordingly, these deaths were censored at the time of death in the cause-specific Cox analysis of cardiovascular mortality and treated as competing events in the Fine–Gray model.

For the specific analysis of cardiovascular mortality, it was taken into account that death from a non-cardiovascular cause prevents the subsequent occurrence of a cardiovascular death. For this reason, in addition to the cause-specific analysis, cardiovascular mortality was assessed by treating non-cardiovascular death as a competing event. The cumulative incidence of cardiovascular death was also estimated at 1, 5 and 10 years.

### 2.4. Statistical Analysis

Continuous variables were expressed as mean ± standard deviation and categorical variables as frequencies and percentages. Comparisons between men and women were carried out using Student’s *t*-test for continuous variables and the χ^2^ test for categorical variables.

Cumulative all-cause mortality during a follow-up period of up to 10 years was estimated using the Kaplan-Meier method and compared between men and women using the log-rank test. Estimates of cumulative mortality with their 95% confidence intervals were calculated at 1, 5 and 10 years. Between-sex comparisons at these time points were performed using exploratory pointwise Z-tests based on Greenwood standard errors, without adjustment for multiple comparisons. Follow-up was administratively censored at 10 years.

The association between sex and all-cause mortality was assessed using Cox regression models. An unadjusted analysis was performed initially, followed by a multivariable model adjusted for potential baseline confounding factors selected for their clinical relevance: age (expressed in 10-year increments), estimated glomerular filtration rate (expressed in 10 mL/min/1.73 m^2^ increments), NYHA functional class, ischaemic aetiology, diabetes mellitus, current smoking, year of inclusion and presence of baseline neoplasia. The results were expressed as hazard ratios (HR) with their corresponding 95% confidence intervals.

Multivariate analyses were performed using a complete-cases approach, with patients having missing data for any of the included variables excluded from each model.

For cardiovascular mortality, a cause-specific Cox model was used, in which non-cardiovascular deaths were censored at the time of their occurrence.

The proportional hazards assumption of the Cox models was assessed using scaled Schoenfeld residuals. No evidence of non-proportional hazards was observed for sex (*p* = 0.769). Evidence of non-proportionality was observed for age, glomerular filtration rate and NYHA functional class. In a sensitivity analysis that allowed for time-dependent effects for these covariates, the estimate of the association between sex and mortality remained virtually unchanged. In addition, a competing-risk analysis was performed using the Fine–Gray model, with non-cardiovascular death considered as a competing event. The results of this model were expressed as subdistribution hazard ratios (sHR) with 95% confidence intervals. The cumulative incidence of cardiovascular mortality was estimated using cumulative incidence functions (CIFs), and differences between men and women were assessed using the Gray test. The assumption of proportional subdistribution hazards in the Fine–Gray model was evaluated using model residuals and an analysis of time-dependent effects of covariates. Where evidence of non-proportionality was detected, sensitivity analyses were carried out, allowing the effect of the corresponding covariate to vary over time.

As the inclusion period spanned 2011 to 2023 and encompassed significant changes in the treatment of HFrEF, the year of inclusion was incorporated into the multivariable models. In addition, the possible modification of the association between sex and mortality over the study period was assessed using interaction terms between sex and year of inclusion, for both all-cause mortality and cardiovascular mortality.

A sensitivity analysis was also carried out by incorporating into the multivariable model for all-cause mortality the four main pharmacological classes known to modify the prognosis of HFrEF recorded at the start of follow-up (beta-blockers, ACEi/ARB/ARNI, MRA and SGLT2i), with the aim of assessing whether their inclusion altered the association between sex and mortality.

Additional analyses assessed a sex-by-ischaemic-aetiology interaction in the multivariable Cox model for all-cause mortality. Separate sensitivity analyses evaluated the robustness of the association between sex and all-cause mortality by: (i) replacing continuous year of inclusion with two calendar periods (2011–2016 and 2017–2023); (ii) additionally adjusting for referral following hospitalisation; (iii) restricting the analysis to patients enrolled between 2011 and 2014; and (iv) additionally adjusting for baseline LVEF and atrial fibrillation.

Competing-risk analyses using the Fine–Gray method and graphical representations of mortality were performed using R, whilst the remaining statistical analyses were carried out using IBM SPSS Statistics version 19.0 (IBM, Armonk, NY, USA). A two-sided *p*-value of <0.05 was considered statistically significant.

### 2.5. Ethical Particulars

The study was approved by the Galician Research Ethics Committee for Medicines (Comité de Ética de la Investigación con Medicamentos de Galicia, CEIm-G; approval code 2025/378) in October 2025, before data extraction and analysis. Given the retrospective observational design and the use of routinely collected clinical data from Hospital Universitario Lucus Augusti covering the period from 2011 to 2023, the ethics committee waived the requirement for individual written informed consent. All data were anonymized before analysis to protect patient confidentiality and ensure compliance with applicable data protection regulations.

## 3. Results

### 3.1. Cohort Description by Sex

The cohort comprised 1510 patients with HFrEF, of whom 1158 (76.7%) were men and 352 (23.3%) were women. The baseline characteristics of the population by sex are presented in Table 1. The overall mean age was 65.9 ± 10.9 years. Women were older than men (68.2 ± 10.8 versus 65.2 ± 10.9 years; *p* < 0.001) and had a lower estimated glomerular filtration rate (65.1 ± 31.1 versus 81.3 ± 33.6 mL/min/1.73 m^2^; *p* < 0.001). No differences were observed in body mass index (28.9 ± 6.8 versus 29.2 ± 8.1 kg/m^2^; *p* = 0.66).

The aetiology of heart failure differed by sex (*p* < 0.001), with ischaemic and alcohol-related aetiologies being more common in men, whilst idiopathic and other aetiologies were more common in women. Baseline left ventricular ejection fraction (LVEF) was comparable between the two sexes (30.6 ± 6.9% in men versus 30.9 ± 6.7% in women; *p* = 0.47).

There were also differences in NYHA functional class (*p* = 0.002). NYHA class I was present in 31.6% of men and 27.6% of women, class II in 63.1% and 61.8%, respectively, and classes III–IV in 5.3% of men and 10.5% of women. Men were more frequently referred to the specialist Heart Failure Unit following hospitalisation than women (74.0% vs. 66.2%; *p* = 0.004).

With regard to cardiovascular risk factors, diabetes mellitus was more common in men (41.3% versus 27.9%; *p* < 0.001), as was current smoking (10.7% versus 4.0%; *p* < 0.001). No significant differences were observed in high blood pressure (68.5% versus 64.5%; *p* = 0.16) or in dyslipidaemia (66.4% versus 66.7%; *p* = 0.92).

Men were more likely to have respiratory comorbidities (13.3% versus 6.8%; *p* < 0.001) and liver disease (8.5% versus 3.7%; *p* = 0.003). There were no significant differences in baseline neoplasia (19.9% versus 22.7%; *p* = 0.26), cognitive impairment (0.6% versus 1.1%; *p* = 0.30) or autoimmune disease (1.7% versus 2.6%; *p* = 0.32). Atrial fibrillation was present in 33.0% of men and 27.2% of women (*p* = 0.05).

### 3.2. Baseline Pharmacological Treatment and Temporal Distribution of the Cohort

No differences were observed between men and women in the main pharmacological classes used for the treatment of HFrEF (Table 2). Beta-blockers were used in 96.5% of men and 96.9% of women (*p* = 0.71), ACEi/ARB/ARNI in 97.0% and 96.6%, respectively (*p* = 0.71), SGLT2 inhibitors in 28.6% and 25.0% (*p* = 0.19), and MRA in 61.6% of both groups (*p* = 0.98). No significant differences were observed in the use of loop diuretics either (55.0% versus 59.7%; *p* = 0.12).

In contrast, treatment with statins was more common in men (55.2% versus 48.3%; *p* = 0.02), as was anticoagulant therapy (38.1% versus 31.8%; *p* = 0.03).

The distribution of men and women by year of inclusion, between 2011 and 2023, showed no significant differences (*p* = 0.24).

### 3.3. Follow-Up and All-Cause Mortality

Information on survival status was available for 1504 of the 1510 patients included: 1153 men and 351 women. The study population and analytical samples for the primary models are summarised in Supplementary Figure S1. The overall mean follow-up was 1775.8 ± 1273.2 days. The mean follow-up was 1749.3 ± 1257.8 days for men and 1863.0 ± 1320.7 days for women, with no statistically significant differences between the two groups (*p* = 0.154). Across the entire available follow-up, 417 patients died. Of these, 172 deaths (41.2%) were classified as cardiovascular and 245 (58.8%) were non-cardiovascular or of unknown cause, including 45 deaths of unknown cause (33 men [73.3%] and 12 women [26.7%]).

No statistically significant difference in cumulative all-cause mortality was observed between men and women at one year: 3.7% (95% CI 2.6–4.8) in men and 3.8% (95% CI 1.7–5.8) in women (*p* = 0.95). At 5 years, the cumulative mortality estimate was 24.8% (95% CI 21.7–27.8) in men and 16.0% (95% CI 11.3–20.6) in women (*p* = 0.002), and at 10 years, 49.3% (95% CI 44.5–53.7) and 39.8% (95% CI 31.1–47.4), respectively (*p* = 0.046).

**Figure 1:**
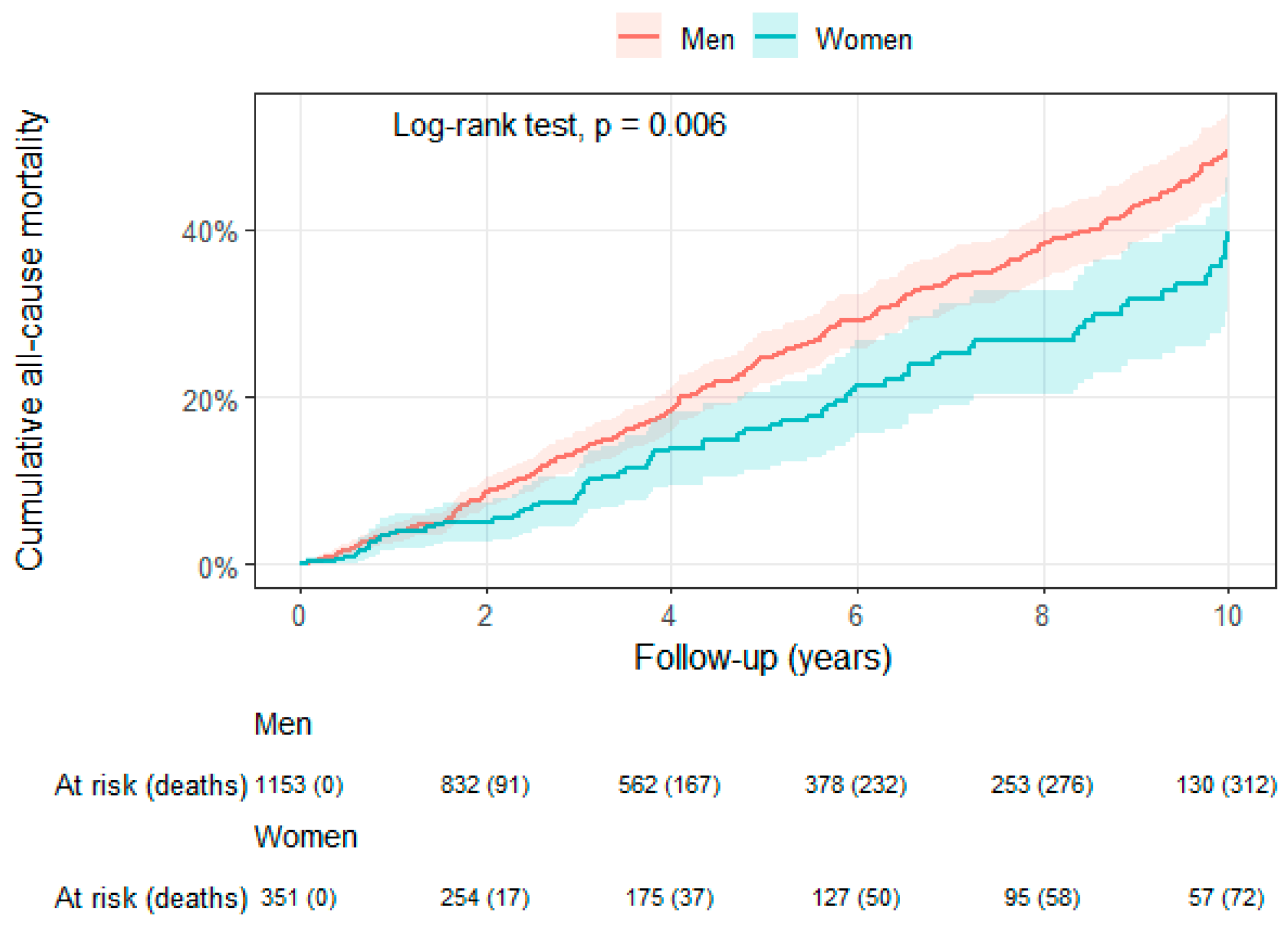
Cumulative all-cause mortality by sex. Kaplan–Meier curves showing cumulative all-cause mortality in men and women during a follow-up period of up to 10 years. Shaded areas represent 95% confidence intervals. Differences between the two groups were assessed using the log-rank test (*p* = 0.006). The numbers below the figure indicate the number of patients at risk and, in brackets, the cumulative number of deaths at each time point. Follow-up was administratively censored at 10 years.

The Kaplan–Meier curves showed a progressive divergence during follow-up, with higher cumulative mortality in men; the overall comparison using the log-rank test was significant (*p* = 0.006; Figure 1).

In the unadjusted analysis, male sex was associated with higher all-cause mortality [HR 1.43 (95% CI 1.11–1.85)]. After adjustment for potential baseline confounding factors, this association became more pronounced and remained statistically significant [HR 1.953 (95% CI 1.474–2.587); *p* < 0.001]. Furthermore, older age, poorer NYHA functional class, ischaemic aetiology, diabetes mellitus and baseline neoplasm were associated with higher mortality, whilst a higher glomerular filtration rate showed an inverse association. Current smoking and year of inclusion did not reach statistical significance (Table 3). The multivariable Cox analysis included 1404 patients (93.4% of those with available survival data). Missing covariate data were primarily observed for estimated glomerular filtration rate (3.9%), diabetes mellitus (1.9%), and current smoking (1.2%). Overall, 100 patients (6.6%) were excluded due to incomplete covariate data, including 71 men (6.2% of men) and 29 women (8.3% of women).

No significant interaction was observed between sex and ischaemic aetiology (HR for interaction 1.07, 95% CI 0.61–1.89; *p* = 0.80). The association between male sex and all-cause mortality remained consistent across sensitivity analyses adjusting for therapeutic periods (HR 1.95, 95% CI 1.47–2.58), incorporating referral from hospitalization (HR 1.95, 95% CI 1.48–2.59), restricting the cohort to patients enrolled between 2011 and 2014 (*n* = 484; HR 2.31, 95% CI 1.61–3.31; Supplementary Table S1) and additionally adjusting for baseline LVEF and atrial fibrillation (*n* = 1291; HR 1.97, 95% CI 1.47–2.64; Supplementary Table S2); all *p* < 0.001.

### 3.4. Cardiovascular Mortality by Sex

In the cause-specific Cox model for cardiovascular mortality, conducted in 1404 patients with 151 cardiovascular deaths, male sex was associated with a higher cause-specific hazard of cardiovascular death compared with female sex [HR 2.455 (95% CI 1.536–3.924); *p* < 0.001]. Older age, poorer NYHA functional class and ischaemic aetiology were also associated with an increased risk of cardiovascular death, whilst a higher glomerular filtration rate and a more recent year of inclusion showed inverse associations. The full results of the model are presented in Table 4.

### 3.5. Competing-Risk Analysis of Cardiovascular Mortality

The results were consistent with the cause-specific analysis when non-cardiovascular death was considered a competing event using the Fine–Gray model. The analysis included 1404 patients, with 151 cardiovascular deaths and 220 non-cardiovascular deaths or deaths of unknown cause considered as competing events. Male sex remained associated with cardiovascular mortality after adjustment for measured baseline covariates (sHR 2.12, 95% CI 1.32–3.39; *p* = 0.002) (Table 4).

**Figure 2:**
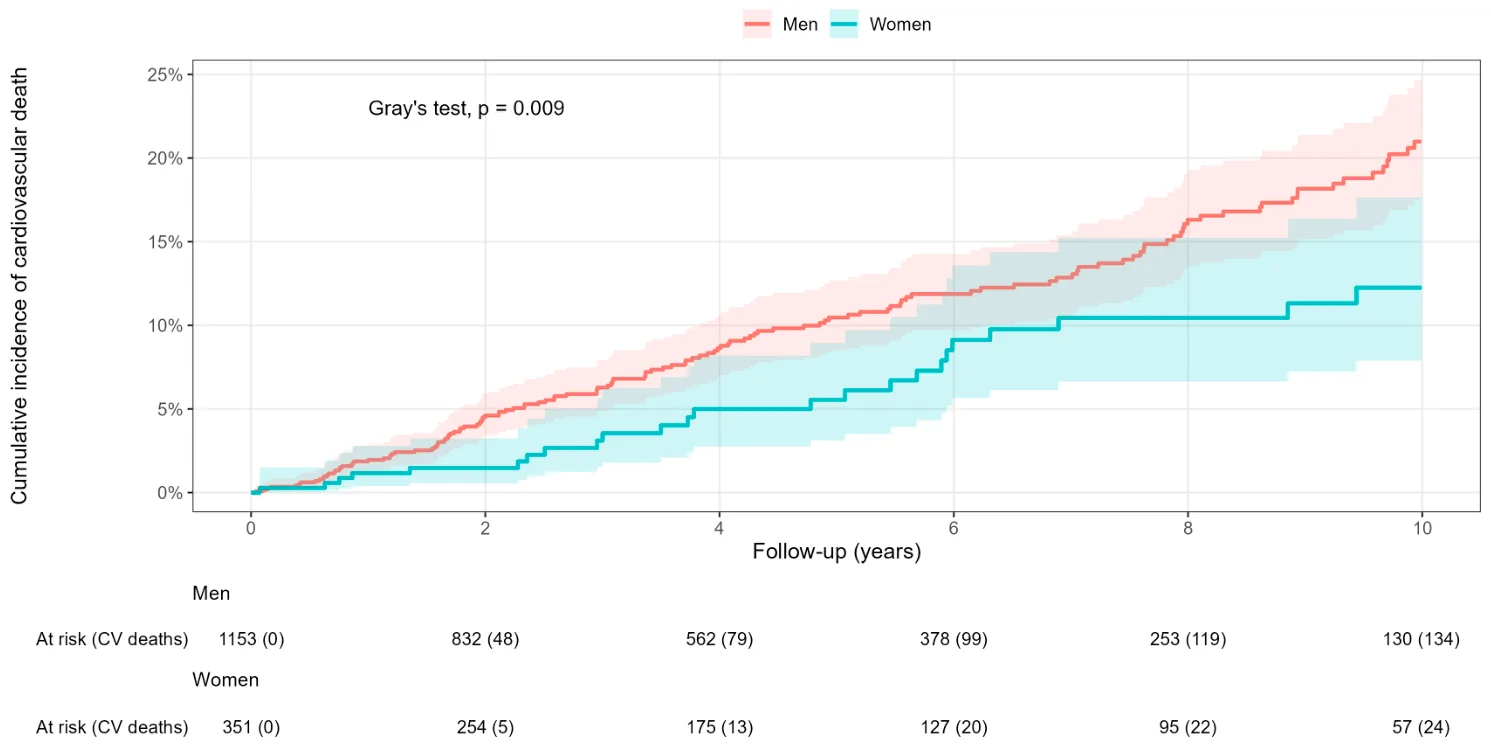
Cumulative incidence of cardiovascular mortality by sex. Cumulative incidence curves for cardiovascular death in men and women, with non-cardiovascular death considered as a competing event. Differences between the two groups were assessed using the Gray test (*p* = 0.009). The numbers below the figure indicate the number of patients at risk and, in brackets, the cumulative number of cardiovascular deaths. Follow-up was administratively censored at 10 years. Shaded areas represent 95% confidence intervals.

The cumulative incidence of cardiovascular death was higher in men throughout the follow-up period. At one year, it was 2.1% (95% CI 1.3–3.1) in men and 1.3% (95% CI 0.4–3.0) in women; at five years, 10.0% (95% CI 8.3–12.0) compared with 5.9% (95% CI 3.3–9.4); and at 10 years, 21% (95% CI 17–25) compared with 13% (95% CI 8.1–18), respectively. The cumulative incidence curves differed significantly by sex according to Gray’s test (*p* = 0.009; Figure 2).

### 3.6. Sensitivity Analysis and Temporal Trends

Given the long inclusion period of the cohort, we assessed whether the association between sex and mortality varied according to the year of inclusion. No statistically significant interaction was observed between sex and year for all-cause mortality (interaction *p* = 0.161) or for cardiovascular mortality (interaction *p* = 0.235), with no evidence of a modification of the association according to the year of inclusion.

Furthermore, a sensitivity analysis was conducted by incorporating into the multivariable model for all-cause mortality the four main pharmacological classes known to modify prognosis and recorded at baseline (beta-blockers, ACEi/ARB/ARNI, MRA and SGLT2i). The inclusion of these variables did not substantially alter the observed association, and male sex remained associated with higher all-cause mortality after additional adjustment for baseline pharmacological treatment [HR 1.871 (95% CI 1.410–2.483); *p* < 0.001], compared with an HR of 1.953 (95% CI 1.474–2.587; *p* < 0.001) in the main model.

## 4. Discussion

In this real-world cohort of patients with HFrEF followed in a specialized Heart Failure Unit, male sex remained associated with higher all-cause mortality (adjusted HR, 1.95; 95% CI, 1.47–2.59) and cardiovascular mortality (cause-specific HR, 2.46; 95% CI, 1.54–3.92) after adjusting for measured baseline covariates. This association with cardiovascular mortality remained significant after accounting for non-cardiovascular death and death of unknown cause as competing events using the Fine–Gray model (subdistribution HR, 2.12; 95% CI, 1.32–3.39). The direction of these findings is consistent with previous evidence from clinical cohorts, randomized trials and meta-analyses reporting a more favorable prognosis among women with HFrEF [6,10,18,19,20,21]. Among the studies most directly comparable with our population, Dewan et al. reported lower all-cause and cardiovascular mortality in women with HFrEF after multivariable adjustment [10], whereas Keshvani et al. found lower adjusted 5-year mortality among women, particularly in patients with HFrEF [21]. However, the association between sex and mortality has not been consistent across all populations, and some cohorts have not identified sex as an independent predictor after multivariable adjustment [22]. The present findings should therefore be interpreted as associations within this clinical population rather than evidence that sex itself causally determines mortality.

The magnitude of the association observed in our cohort was greater than that reported in several large series and meta-analyses. Expressed as hazard ratios for mortality in men compared with women, the corresponding estimates were approximately 1.47 in the analysis by Dewan et al. of the PARADIGM-HF and ATMOSPHERE trials, which included exclusively patients with HFrEF and incorporated NT-proBNP into the adjustment model [10], approximately 1.20 in the meta-analysis by Qiu et al. including more than 700,000 patients [12], and approximately 1.56 in CIBIS-II [19]. Our point estimates for all-cause mortality (1.95) and cardiovascular mortality (2.46) were higher than those reported in these populations. Nevertheless, differences in study populations, clinical characteristics and adjustment strategies, together with the uncertainty surrounding our estimates, preclude direct conclusions regarding the relative strength of these associations across studies. In particular, the absence of systematic adjustment for natriuretic peptides, device therapy and longitudinal treatment exposure may have contributed to the magnitude of the associations observed in our cohort.

Cumulative all-cause mortality was higher in men during long-term follow-up. Exploratory pointwise comparisons detected differences at five and ten years, but not at one year. The absence of a statistically significant difference at one year does not establish equivalent early mortality, and differences in statistical significance across follow-up time points do not demonstrate a change in the relative association between sex and mortality. The proportional hazards assessment did not detect evidence of non-proportionality for sex (*p* = 0.769). Thus, the progressive separation of cumulative mortality curves should not be interpreted as evidence of an increasing hazard ratio over time. These observations can be considered alongside the meta-analysis by Qiu et al., in which the lower mortality observed among women was more pronounced in chronic heart failure and in studies with follow-up exceeding two years [12]. Similarly, Klempfner et al. found no significant difference in mortality between men and women during the first six months after hospitalization for heart failure, whereas male sex was associated with higher mortality during subsequent follow-up [13]. However, these studies involved different populations and analytical approaches and do not establish a comparable time-varying association in our cohort. Moreover, the pointwise comparisons in the present study were exploratory and were not adjusted for multiple comparisons; the ten-year comparison requires particular caution because of the smaller numbers remaining at risk.

Baseline differences between men and women may have contributed to the observed mortality patterns. In our cohort, women were older and had poorer renal function and worse NYHA functional class, whereas men more frequently had an ischaemic aetiology, diabetes mellitus and current smoking. This overall pattern is consistent with previous studies of HFrEF, in which women generally have a lower burden of ischaemic disease despite presenting with other adverse clinical characteristics [6,10,18]. In addition, the MAGGIC meta-analysis showed that the prognostic advantage associated with female sex was more pronounced in patients with non-ischaemic aetiology and in those without diabetes [20]. In our cohort, the association between male sex and mortality persisted after adjustment for ischaemic aetiology, diabetes and other measured baseline factors. No statistically significant sex-by-ischaemic-aetiology interaction was detected, although the uncertainty around the interaction estimate does not exclude clinically relevant effect modification. Notably, the magnitude of the association increased after multivariable adjustment. This finding suggests that adverse characteristics that were more prevalent among women, particularly older age, poorer renal function and worse functional class, may have partially attenuated the association between male sex and mortality in unadjusted analyses. This explanation remains an interpretation of the observed adjustment pattern rather than a demonstrated causal mechanism.

Cause-specific Cox and Fine–Gray models provided complementary perspectives on the association between male sex and cardiovascular mortality. The cause-specific model showed that, among patients who remained alive, men had a higher instantaneous rate of cardiovascular mortality (HR, 2.46). The Fine–Gray model identified an association between male sex and the subdistribution hazard of cardiovascular death (sHR, 2.12), accounting for non-cardiovascular deaths and deaths of unknown cause as competing events. These estimates describe different quantities and should not be interpreted as interchangeable measures of absolute risk. Consistently, cumulative incidence curves for cardiovascular death differed between sexes. Taken together, these findings show that the association was observed using complementary analytical approaches, although both remain subject to the same limitations of the observational data and cause-of-death classification.

The association between male sex and cardiovascular mortality raises the possibility that sex-related differences in the mechanisms underlying cardiovascular death may contribute to the observed prognostic disparity. Previous studies have reported lower risks of sudden cardiac death and malignant ventricular arrhythmias among women with HFrEF [10,23,24]. Rho et al. observed a lower risk of sudden cardiac death among women with heart failure who were candidates for primary-prevention implantable cardioverter-defibrillator therapy [23], whereas Saxena et al. reported a lower incidence of sustained ventricular tachyarrhythmias among women with implantable cardioverter-defibrillators [24]. These findings provide a rationale for considering arrhythmic susceptibility as a potential contributor to sex-related differences in cardiovascular mortality. However, the present analysis did not examine specific modes of cardiovascular death or systematically account for device therapy. It therefore cannot determine whether arrhythmic mechanisms explain the observed association.

Differences in the evolution of ventricular function may represent another potential contributor to sex-related differences in long-term prognosis. Previous studies have reported a greater likelihood of LVEF recovery among women with HFrEF [25,26,27,28], while our previous analysis of this cohort identified follow-up LVEF as an important prognostic factor [2]. However, the present study was not designed to evaluate whether ventricular recovery contributes to the association between sex and mortality. Furthermore, follow-up echocardiographic assessments were subject to survivor and selection bias, as they were only available for patients who survived long enough to undergo reassessment. Therefore, no conclusions can be drawn regarding the potential mediating role of ventricular reverse remodelling in the observed sex-related mortality differences.

Baseline use of the major guideline-directed medical therapy classes did not differ significantly between men and women. Moreover, in a sensitivity analysis incorporating beta-blockers, ACEi/ARB/ARNI, mineralocorticoid receptor antagonists and SGLT2 inhibitors into the multivariable model, male sex remained associated with higher mortality (HR, 1.87; 95% CI, 1.41–2.48; *p* < 0.001), with an estimate closely resembling that of the primary model. These findings indicate that additional adjustment for recorded baseline treatment classes did not materially alter the association. They do not establish equivalent treatment exposure over time or exclude differences in dose, titration, adherence, discontinuation or access to device therapy. Meta-analyses and analyses of major HFrEF trials have not demonstrated a consistent interaction between sex and the treatment effect of the principal guideline-directed therapies [29,30,31]. However, treatment efficacy in clinical trials and treatment delivery in routine practice are distinct questions, and the present data cannot determine the contribution of longitudinal treatment differences to mortality.

The present study extends our previous investigation of early and late mortality predictors in the same cohort [2] by focusing on sex-related differences and adding an assessment of cardiovascular mortality using competing-risk regression. Its incremental contribution lies in this dedicated analysis and the complementary evaluation of cause-specific and subdistribution hazards, rather than in demonstrating a new time-dependent effect of sex. Overall, male sex remained associated with higher all-cause and cardiovascular mortality after adjustment for measured baseline covariates. The analyses cannot establish how much of these associations reflects biological differences, disease phenotype, treatment trajectories, healthcare access or other unmeasured factors. These findings require external validation before their incremental value for clinical risk prediction can be determined.

## 5. Strengths and Limitations

The strengths of this study include the consecutive enrolment of patients managed at a specialized heart failure clinic, follow-up extending up to ten years, and multivariable adjustment for several measured baseline prognostic factors. Building on our previous analysis of early and late mortality predictors in this HFrEF cohort [2], the present study provides a dedicated assessment of sex-related differences in long-term all-cause and cardiovascular mortality using complementary cause-specific and competing-risk models.

Several limitations should be considered. First, the retrospective, single-centre observational design limits generalizability and precludes causal inference. Although adjustment and sensitivity analyses yielded consistent findings, residual confounding remains possible. Natriuretic peptide concentrations, device therapy and longitudinal treatment exposure were not systematically incorporated into the models. Gender identity and sociocultural characteristics were not collected, and differences in healthcare access, disease management and treatment adherence may also have contributed to the observed associations. The findings should therefore be interpreted as associations after adjustment for measured baseline covariates rather than causal effects of sex.

Second, the primary multivariable analyses used a complete-case approach, excluding 100 of the 1504 patients with available survival information. The proportion excluded was 6.2% among men and 8.3% among women. If missingness was related to prognosis or other patient characteristics, these exclusions may have introduced selection bias.

Third, although additional adjustment for referral following hospitalisation did not materially alter the association, referral setting does not capture the duration of heart failure before cohort entry. Men and women may have entered the specialist unit at different stages of their disease, and potential lead-time bias cannot be excluded.

Fourth, retrospective classification of cause of death remains susceptible to misclassification despite review of the available clinical documentation. Deaths of unknown cause were grouped with non-cardiovascular deaths as competing events. Some of these deaths may have been cardiovascular, potentially affecting the estimates of cardiovascular mortality and its association with sex.

Fifth, women represented a minority of the cohort, and the relatively small number of cardiovascular deaths among women limited statistical precision. The exact magnitude of the sex-related associations should therefore be interpreted cautiously.

Sixth, ten-year estimates require caution because of the progressive decline in the number of patients remaining at risk, with 130 men and 57 women at ten years. Only patients enrolled sufficiently early could contribute follow-up approaching ten years, so these estimates principally reflect earlier inclusion periods. Furthermore, comparisons at one, five and ten years were exploratory and were not adjusted for multiple comparisons. Differences in significance across these time points do not establish a time-varying association between sex and mortality.

Seventh, the prolonged inclusion period encompassed substantial changes in HFrEF management, including the introduction of ARNIs and SGLT2 inhibitors. Changes in treatment practice have been described in a previous study from our centre involving a largely overlapping cohort [32]. Adjustment for year of inclusion and a sensitivity analysis using calendar periods yielded consistent results. Nevertheless, treatment information in the present analysis was restricted to baseline prescriptions and did not capture subsequent dose titration, adherence, discontinuation or treatment changes. Adjustment for baseline treatment classes therefore cannot exclude a contribution from differences in treatment exposure or optimisation during follow-up.

Eighth, the classification of deaths of unknown cause as competing events warrants consideration. If some of these deaths were actually cardiovascular, their classification as competing events rather than cardiovascular deaths could have led to an underestimation of the cumulative incidence of cardiovascular mortality. Furthermore, the potential impact on the association between sex and cardiovascular mortality would depend, in part, on whether cause-of-death misclassification differed between men and women. Given the retrospective nature of cause-of-death ascertainment, this possibility cannot be excluded.

Finally, the present analyses cannot establish the mechanisms underlying the observed associations. Although differences in ventricular remodelling and arrhythmic susceptibility are plausible contributors, these pathways were not directly evaluated. Follow-up echocardiographic measurements were subject to survivor and selection bias, as they were available only in patients who survived and underwent reassessment. Consequently, no conclusions can be drawn regarding the mediating role of ventricular recovery or specific modes of cardiovascular death.

## 6. Conclusions

In this single-centre observational cohort of patients with HFrEF, male sex remained associated with higher all-cause and cardiovascular mortality after adjustment for measured baseline covariates. The association with cardiovascular mortality was observed in both cause-specific and Fine–Gray models. Differences in cumulative all-cause mortality were detected at five and ten years in exploratory comparisons, but not at one year; this does not establish a time-varying effect of sex. Residual confounding, incomplete covariate data and uncertainty in late follow-up estimates should be considered when interpreting these findings.

## Figures and Tables

**Table 1:** Baseline clinical characteristics by sex.

*p*-Value	Women (*n* = 352)	Men (*n* = 1158)	*N*	Total (*n* = 1510)	Variable
-	-	-	1510	1158 (76.7)	Male sex
<0.001	68.2 ± 10.8	65.2 ± 10.9	1509	65.9 ± 10.9	Age, years
0.66	28.9 ± 6.8	29.2 ±8.1	1456	29.1 ± 7.8	BMI, kg/m^2^
<0.001	65.1 ± 31.1	81.3 ± 33.6	1452	77.5 ± 33.7	eGFR, mL/min/1.73 m^2^
eGFR category, mL/min/1.73 m^2^					
<0.001	58 (17.2)	406 (36.4)	1452	464 (32)	GFR ≥ 90
	110 (32.5)	401 (36)		511 (35.2)	GFR 60–89.9
	144 (42.6)	275 (24.7)		419 (28.9)	GFR 30–59.9
	22 (6.5)	27 (2.4)		49 (3.4)	GFR 15–29.9
	4 (1.2)	5 (0.4)		9 (0.6)	GFR < 15
Primary etiology					
<0.001	66 (18.8)	498 (43.0)	1510	564 (37.4)	Ischemic
	122 (34.7)	186 (16.1)		308 (20.4)	Idiopathic
	43 (12.2)	119 (10.3)		162 (10.7)	Arrhythmic
	2 (0.6)	135 (11.7)		137 (9.1)	Alcohol-related
	43 (12.2)	89 (7.7)		132 (8.7)	Hypertensive
	76 (21.6)	131 (11.3)		207 (13.7)	Other
0.002	97 (27.6)	363 (31.6)	1501	460 (30.6)	NYHA I
	217 (61.8)	726 (63.1)		943 (62.8)	NYHA II
	37 (10.5)	61 (5.3)		98 (6.5)	NYHA III or IV
0.004	233 (66.2)	857 (74.0)	1510	1090 (72.2)	Referral from hospitalization
Cardiovascular risk factors					
0.16	223 (64.5)	784 (68.5)	1491	1007 (67.5)	Hypertension
<0.001	95 (27.9)	471 (41.3)	1481	566 (38.2)	Diabetes
0.92	228 (66.7)	758 (66.4)	1484	986 (66.4)	Dyslipidemia
<0.001	14 (4)	122 (10.7)	1491	136 (9.1)	Active smoker
Comorbidities					
<0.001	24 (6.8)	154 (13.3)	1510	178 (11.8)	Respiratory
0.26	80 (22.7)	231 (19.9)	1510	311 (20.6)	Cancer
0.30	4 (1.1)	7 (0.6)	1510	11 (0.7)	Cognitive decline
0.003	13 (3.7)	98 (8.5)	1510	111 (7.4)	Hepatic disease
0.32	9 (2.6)	20 (1.7)	1510	29 (1.9)	Autoimmune disease
0.05	87 (27.2)	343 (33)	1360	430 (31.6)	Atrial fibrillation

Data presented as mean ± standard deviation or *n* (%), as appropriate. *N* indicates the number of patients with available data for each variable. BMI, body mass index; eGFR, estimated glomerular filtration rate; LVEF, left ventricular ejection fraction; CKD, chronic kidney disease; NYHA, New York Heart Association.

**Table 2:** Baseline pharmacological treatment.

*p*-Value	Women (*n* = 352)	Men (*n* = 1158)	Total (*n* = 1510)	Variable
0.71	341 (96.9)	1117 (96.5)	1458 (96.6)	Beta-blockers
0.71	340 (96.6)	1123 (97)	1463 (96.9)	ACEi/ARB/ARNI
0.19	88 (25)	331 (28.6)	419 (27.7)	SGLT2i
0.98	217 (61.6)	713 (61.6)	930 (61.6)	MRA
0.12	210 (59.7)	637 (55)	847 (56.1)	Loop diuretic
0.02	170 (48.3)	639 (55.2)	809 (53.6)	Statins
0.03	112 (31.8)	441 (38.1)	553 (36.6)	Anticoagulant

Data presented as *n* (%). ACEi, angiotensin-converting enzyme inhibitor; ARB, angiotensin receptor blocker; ARNI, angiotensin receptor-neprilysin inhibitor; MRA, mineralocorticoid receptor antagonist; SGLT2, sodium-glucose cotransporter 2. Data are presented as *n* (%).

**Table 3:** Multivariable Cox regression analysis for all-cause mortality.

*p*-Value	HR (95% CI)	Variable
<0.001	1.953 (1.474–2.587)	Male sex
<0.001	1.403 (1.216–1.620)	Age, per 10 years
<0.001	0.766 (0.693–0.847)	eGFR, per 10 mL/min/1.73 m^2^
<0.001	1.447 (1.203–1.740)	NYHA functional class per category increase
0.034	1.255 (1.017–1.549)	Ischemic etiology
0.008	1.322 (1.075–1.625)	Diabetes mellitus
0.082	1.420 (0.957–2.108)	Active smoking
0.131	0.971 (0.935–1.009)	Year of inclusion
<0.001	1.599 (1.288–1.986)	Baseline neoplasia

HR, hazard ratio; CI, confidence interval; eGFR, estimated glomerular filtration rate; NYHA, New York Heart Association. Age and eGFR are expressed per 10-year and per 10 mL/min/1.73 m^2^ increments, respectively. NYHA functional class was coded as I, II, and III/IV. Multivariable Cox analysis: *n* = 1404; 371 deaths.

**Table 4:** Cause-specific Cox and Fine–Gray analyses for cardiovascular mortality.

*p*-Value	Fine–Gray sHR (95% CI)	*p*-Value	Cause-Specific HR (95% CI)	Variable
0.002	2.12 (1.32–3.39)	<0.001	2.455 (1.536–3.924)	Male sex
<0.001	1.57 (1.25–1.97)	<0.001	1.665 (1.320–2.098)	Age, per 10 years
0.030	0.83 (0.71–0.98)	0.006	0.811 (0.698–0.943)	eGFR, per 10 mL/min/1.73 m^2^
0.002	1.62 (1.20–2.20)	<0.001	1.741 (1.310–2.314)	NYHA functional class per category increase
<0.001	2.17 (1.54–3.06)	<0.001	2.208 (1.569–3.108)	Ischemic etiology
0.16	1.27 (0.91–1.76)	0.059	1.366 (0.988–1.888)	Diabetes mellitus
0.86	1.07 (0.50–2.29)	0.726	1.148 (0.530–2.488)	Active smoking
<0.001	0.90 (0.85–0.95)	0.005	0.917 (0.863–0.974)	Year of inclusion
<0.001	0.39 (0.24–0.64)	0.001	0.424 (0.261–0.688)	Baseline neoplasia

HR, hazard ratio; sHR, subdistribution hazard ratio; CI, confidence interval; eGFR, estimated glomerular filtration rate; NYHA, New York Heart Association. Age and eGFR are expressed per 10-year and per 10 mL/min/1.73 m^2^ increments, respectively. NYHA functional class was coded as I, II, and III/IV. Cause-specific Cox analysis: *n* = 1404, 151 cardiovascular deaths. Fine–Gray analysis: *n* = 1404, 151 cardiovascular deaths and 220 non-cardiovascular deaths or deaths of unknown cause considered as competing events. The proportional subdistribution hazards assumption was met for sex; a time-varying effect was observed for NYHA, and sensitivity analysis did not materially alter the association between sex and cardiovascular mortality.

## Data Availability

The original contributions presented in this study are included in the article. Further inquiries can be directed to the corresponding authors.

## Supplementary Materials

The following supporting information can be downloaded at: https://www.mdpi.com/article/10.3390/jcm15218431/s1, Figure S1: Flow diagram of the study population and primary analytical samples; Table S1: Multivariable Cox regression analysis of all-cause mortality in patients enrolled between 2011 and 2014; Table S2: Sensitivity analysis of all-cause mortality additionally adjusted for baseline LVEF and atrial fibrillation.

## References

[1] Lund L.H., Maggioni A.P., Crespo-Leiro M.G., Laroche C., Gotsman I., Pojskic B., Vataman E.B., Grigorica L., Sisakian H., Glavas D. (2026). Outcomes of heart failure with reduced, mildly reduced, or preserved ejection fraction: The ESC HF III registry. Eur. Heart J..

[2] Lopez-Lopez A., Vázquez E.R., Regueiro-Abel M., Álvarez-Suárez C.C., Johk-Casas C.A., Elices-Teja J., Vázquez R.R., Santamarina-Pernas G., González-Juanatey C. (2026). Temporal trends and predictors of mortality in heart failure with reduced ejection fraction: The role of renal function and left ventricular improvement. J. Clin. Med..

[3] Whitelaw S., Sullivan K., Eliya Y., Alruwayeh M., Thabane L., Yancy C.W., Mehran R., Mamas M.A., Van Spall H.G. (2021). Trial characteristics associated with under-enrolment of females in randomized controlled trials of heart failure with reduced ejection fraction: A systematic review. Eur. J. Heart Fail..

[4] Ekpo E., Balla S., Ngo S., Witting C., Sarraju A., Furst A., Rodriguez F. (2024). Underrepresentation of women in reduced ejection heart failure clinical trials with improved mortality or hospitalization. JACC Adv..

[5] O’MEara E., Clayton T., McEntegart M.B., McMurray J.J., Piña I.L., Granger C.B., Östergren J., Michelson E.L., Solomon S.D., Pocock S. (2007). Sex differences in clinical characteristics and prognosis in a broad spectrum of patients with heart failure: Results of the Candesartan in Heart Failure: Assessment of Reduction in Mortality and Morbidity (CHARM) program. Circulation.

[6] Stolfo D., Uijl A., Vedin O., Strömberg A., Faxén U.L., Rosano G.M., Sinagra G., Dahlström U., Savarese G. (2019). Sex-based differences in heart failure across the ejection fraction spectrum: Phenotyping, and prognostic and therapeutic implications. JACC Heart Fail..

[7] Lam C.S.P., Arnott C., Beale A.L., Chandramouli C., Hilfiker-Kleiner D., Kaye D.M., Ky B., Santema B.T., Sliwa K., Voors A. (2019). Sex differences in heart failure. Eur. Heart J..

[8] Martin T.G., Leinwand L.A. (2024). Hearts apart: Sex differences in cardiac remodeling in health and disease. J. Clin. Investig..

[9] Sumarsono A., Xie L., Keshvani N., Zhang C., Patel L., Alonso W.W., Thibodeau J.T., Fonarow G.C., Van Spall H.G., Messiah S.E. (2024). Sex disparities in longitudinal use and intensification of guideline-directed medical therapy among patients with newly diagnosed heart failure with reduced ejection fraction. Circulation.

[10] Dewan P., Rørth R., Jhund P.S., Shen L., Raparelli V., Petrie M.C., Abraham W.T., Desai A.S., Dickstein K., Køber L. (2019). Differential impact of heart failure with reduced ejection fraction on men and women. J. Am. Coll. Cardiol..

[11] Kim H.M., Kim H.L., Kim M.A., Lee H.Y., Park J.J., Choi D.J. (2024). Sex differences in clinical characteristics and long-term outcome in patients with heart failure: Data from the KorAHF registry. Korean J. Intern. Med..

[12] Qiu W., Wang W., Wu S., Zhu Y., Zheng H., Feng Y. (2024). Sex differences in long-term heart failure prognosis: A comprehensive meta-analysis. Eur. J. Prev. Cardiol..

[13] Klempfner R., Koifman E., Goldenberg I., Hamdan A., Tofler G.H., Kopel E. (2014). The Israel Nationwide Heart Failure Survey: Sex differences in early and late mortality for hospitalized heart failure patients. J. Card. Fail..

[14] Santas E., Palau P., Llácer P., de la Espriella R., Miñana G., Núñez-Marín G., Lorenzo M., Heredia R., Sanchis J., Chorro F.J. (2022). Sex-related differences in mortality following admission for acute heart failure across the left ventricular ejection fraction spectrum. J. Am. Heart Assoc..

[15] Taylor C.J., Ordóñez-Mena J.M., Jones N.R., Roalfe A.K., Lay-Flurrie S., Marshall T., Hobbs F.R. (2021). National trends in heart failure mortality in men and women, United Kingdom, 2000–2017. Eur. J. Heart Fail..

[16] Settergren C., Benson L., Shahim A., Dahlström U., Thorvaldsen T., Savarese G., Lund L.H., Shahim B. (2024). Cause-specific death in heart failure across the ejection fraction spectrum: A comprehensive assessment of over 100,000 patients in the Swedish Heart Failure Registry. Eur. J. Heart Fail..

[17] Gregson J., Pocock S.J., Anker S.D., Bhatt D.L., Packer M., Stone G.W., Zeller C. (2024). Competing risks in clinical trials: Do they matter and how should we account for them?. J. Am. Coll. Cardiol..

[18] Pabon M., Cunningham J., Claggett B., Felker G.M., McMurray J.J., Metra M., Diaz R., Wang X., Arias-Mendoza A., Bonderman D. (2023). Sex differences in heart failure with reduced ejection fraction in the GALACTIC-HF trial. JACC Heart Fail..

[19] Simon T., Mary-Krause M., Funck-Brentano C., Jaillon P. (2001). Sex differences in the prognosis of congestive heart failure: Results from the Cardiac Insufficiency Bisoprolol Study (CIBIS II). Circulation.

[20] Martínez-Sellés M., Doughty R.N., Poppe K., Whalley G.A., Earle N., Tribouilloy C., McMurray J.J., Swedberg K., Køber L., Berry C. (2012). Gender and survival in patients with heart failure: Interactions with diabetes and aetiology. Results from the MAGGIC individual patient meta-analysis. Eur. J. Heart Fail..

[21] Keshvani N., Shah S., Ayodele I., Chiswell K., Alhanti B., Allen L.A., Greene S.J., Yancy C.W., Alonso W.W., Van Spall H.G. (2023). Sex differences in long-term outcomes following acute heart failure hospitalization: Findings from the Get With The Guidelines-Heart Failure registry. Eur. J. Heart Fail..

[22] Mansur A.d.P., Del Carlo C.H., Gonçalinho G.H.F., Avakian S.D., Ribeiro L.C., Ianni B.M., Fernandes F., César L.A.M., Bocchi E.A., Pereira-Barretto A.C. (2022). Sex differences in heart failure mortality with preserved, mildly reduced and reduced ejection fraction: A retrospective, single-center, large-cohort study. Int. J. Environ. Res. Public Health.

[23] Rho R.W., Patton K.K., Poole J.E., Cleland J.G., Shadman R., Anand I., Maggioni A.P., Carson P.E., Swedberg K., Levy W.C. (2012). Important differences in mode of death between men and women with heart failure who would qualify for a primary prevention implantable cardioverter-defibrillator. Circulation.

[24] Saxena S., Goldenberg I., McNitt S., Hsich E., Kutyifa V., Bragazzi N.L., Polonsky B., Aktas M.K., Huang D.T., Rosero S. (2022). Sex differences in the risk of first and recurrent ventricular tachyarrhythmias among patients receiving an implantable cardioverter-defibrillator for primary prevention. JAMA Netw. Open.

[25] Aimo A., Vergaro G., Castiglione V., Barison A., Pasanisi E., Petersen C., Chubuchny V., Giannoni A., Poletti R., Maffei S. (2017). Effect of sex on reverse remodeling in chronic systolic heart failure. JACC Heart Fail..

[26] Kewcharoen J., Trongtorsak A., Thangjui S., Kanitsoraphan C., Prasitlumkum N. (2022). Female gender is associated with an increased left ventricular ejection fraction recovery in patients with heart failure with reduced ejection fraction. Med. Sci..

[27] Ibrahim N.E., Piña I.L., Camacho A., Bapat D., Felker G.M., Maisel A.S., Butler J., Prescott M.F., Abbas C.A., Solomon S.D. (2020). Sex-based differences in biomarkers, health status, and reverse cardiac remodelling in patients with heart failure with reduced ejection fraction treated with sacubitril/valsartan. Eur. J. Heart Fail..

[28] Heredia-Campos G., Rodríguez-Gómez M.E., Perea-Armijo J., González-Manzanares R., Castillo-Domínguez J.C., Delgado-Ortega M., Crespin-Crespin M., Ruiz-Ortiz M., Mesa-Rubio D., Ladera-Santos D. (2025). Heart failure with reduced ejection fraction: Influence of gender on clinical characteristics, cardiac remodeling and neurohormonal response. Med. Clin..

[29] Danielson C., Lileikyte G., Ouwerkerk W., Lam C.S.P., Erlinge D., Teng T.H.K. (2022). Sex differences in efficacy of pharmacological therapies in heart failure with reduced ejection fraction: A meta-analysis. ESC Heart Fail..

[30] Butt J.H., Docherty K.F., Petrie M.C., Schou M., Kosiborod M.N., O’meara E., Katova T., Ljungman C.E.A., Diez M., Ogunniyi M.O. (2021). Efficacy and safety of dapagliflozin in men and women with heart failure with reduced ejection fraction: A prespecified analysis of the Dapagliflozin and Prevention of Adverse Outcomes in Heart Failure trial. JAMA Cardiol..

[31] Zannad F., Ferreira J.P., Pocock S.J., Anker S.D., Butler J., Filippatos G., Brueckmann M., Ofstad A.P., Pfarr E., Jamal W. (2020). SGLT2 inhibitors in patients with heart failure with reduced ejection fraction: A meta-analysis of the EMPEROR-Reduced and DAPA-HF trials. Lancet.

[32] López-López A., Regueiro-Abel M., Johk-Casas C.A., Vieitez-Flórez J.M., Elices-Teja J., Armesto-Rivas J., de Urbano-Seara G., López-Pena A.M., Álvarez-Suárez C.C., Rois-González G. (2026). The impact of introducing sacubitril/valsartan and SGLT2 inhibitors in a cohort of patients with reduced-ejection-fraction heart failure: A real-life observational study. J. Clin. Med..

