# Supplementary material for: Sex Differences in All-Cause and Cardiovascular Mortality During Long-Term Follow-Up in Patients with HFrEF
Source: J Clin Med. 2026 Oct 29;15(21):8431. doi: 10.3390/jcm15218431 (PMC13764507; doi:10.3390/jcm15218431)
Supplement: Supplementary file 1 [file jcm-15-08431-s001.zip › Supplementary figure S1 legend.pdf]

Supplementary Figure S1. Flow diagram of the study population and primary analytical samples.

The initial cohort comprised 1,510 patients (1,158 men and 352 women). Six patients (five men and one woman) were excluded from survival analyses because survival information was unavailable, leaving 1,504 patients (1,153 men and 351 women). Of these, 100 patients (71 men and 29 women) were excluded from the primary multivariable analyses because of incomplete baseline covariate data. The primary complete-case sample comprised 1,404 patients (1,082 men and 322 women) for the all-cause mortality Cox model, the cardiovascular cause-specific Cox model, and the Fine–Gray model. Exclusion percentages were calculated using the number of patients with available survival information within each sex group. CV, cardiovascular.
