# Supplementary material for: Sex Differences in All-Cause and Cardiovascular Mortality During Long-Term Follow-Up in Patients with HFrEF
Source: J Clin Med. 2026 Oct 29;15(21):8431. doi: 10.3390/jcm15218431 (PMC13764507; doi:10.3390/jcm15218431)
Supplement: Supplementary file 1 [file jcm-15-08431-s001.zip › Supplementary table S1.pdf]

**Supplementary Table S1. Multivariable Cox regression analysis of all-cause mortality in patients enrolled between 2011 and 2014**		
**Variable**	**HR (95% CI)**	**p-value**
Male sex	2.31 (1.61–3.31)	<0.001
Age, per 10 years	1.41 (1.17–1.70)	<0.001
eGFR, per 10 mL/min/1.73 m^2	0.76 (0.67–0.87)	<0.001
NYHA functional class per category increase	1.42 (1.14–1.76)	0.002
Ischaemic aetiology	1.43 (1.09–1.87)	0.009
Diabetes mellitus	1.31 (1.01–1.72)	0.045
Active smoking	1.83 (1.10–3.05)	0.020
Year of inclusion	0.94 (0.81–1.10)	0.435
Baseline neoplasia	1.13 (0.84–1.52)	0.413

HR, hazard ratio; CI, confidence interval; eGFR, estimated glomerular filtration rate; NYHA, New York Heart Association. Age and eGFR are expressed per 10-year and per 10 mL/min/1.73 m^2 increments, respectively. NYHA functional class was coded as I, II, and III/IV and modelled per category increase. The analysis included 484 patients and 228 deaths.
