# Supplementary material for: Sex Differences in All-Cause and Cardiovascular Mortality During Long-Term Follow-Up in Patients with HFrEF
Source: J Clin Med. 2026 Oct 29;15(21):8431. doi: 10.3390/jcm15218431 (PMC13764507; doi:10.3390/jcm15218431)
Supplement: Supplementary file 1 [file jcm-15-08431-s001.zip › Supplementary table S2.pdf]

Supplementary Table S2. Sensitivity analysis of all-cause mortality additionally adjusted for baseline LVEF and atrial fibrillation

Variable	Adjusted HR (95% CI)	p-value
Male sex	1.97 (1.47–2.64)	*<0.001*
Age, per 10 years	1.41 (1.21–1.63)	*<0.001*
eGFR, per 10 mL/min/1.73 m^2	0.76 (0.68–0.84)	*<0.001*
NYHA functional class per category increase	1.46 (1.21–1.76)	*<0.001*
Ischaemic aetiology	1.26 (1.01–1.57)	*0.044*
Diabetes mellitus	1.31 (1.06–1.63)	*0.013*
Active smoking	1.30 (0.86–1.98)	0.211
Year of inclusion	0.96 (0.92–1.00)	0.065
Baseline neoplasia	1.57 (1.26–1.97)	*<0.001*
Atrial fibrillation	1.07 (0.86–1.35)	0.535
Baseline LVEF, per 10 percentage-point increase	0.97 (0.84–1.14)	0.760

HR, hazard ratio; CI, confidence interval; eGFR, estimated glomerular filtration rate; NYHA, New York Heart Association; LVEF, left ventricular ejection fraction. Age, eGFR and baseline LVEF are expressed per 10-year, per 10 mL/min/1.73 m^2 and per 10 percentage-point increments, respectively. NYHA functional class was coded as I, II, and III/IV and modelled per category increase. The analysis included 1,291 patients and 351 deaths.
